# Usefulness of Denoising Process to Depict Myopic Choroidal Neovascularisation Using a Single Optical Coherence Tomography Angiography Image

**DOI:** 10.1038/s41598-020-62607-6

**Published:** 2020-04-10

**Authors:** Yuka Sawai, Manabu Miyata, Akihito Uji, Sotaro Ooto, Hiroshi Tamura, Naoko Ueda-Arakawa, Yuki Muraoka, Masahiro Miyake, Ayako Takahashi, Yu Kawashima, Shin Kadomoto, Yasuyuki Oritani, Kentaro Kawai, Kenji Yamashiro, Akitaka Tsujikawa

**Affiliations:** 1 Department of Ophthalmology and Visual Sciences, Kyoto University Graduate School of Medicine, Kyoto, Japan; 2 Department of Ophthalmology, Red Cross Otsu Hospital, Otsu, Japan

**Keywords:** Medical research, Structural biology

## Abstract

Quality of single optical coherence tomography angiography (OCTA) images of myopic choroidal neovascularisation (mCNV) is poorer than in averaged images, although obtaining averaged images takes much time. This study evaluated the clinical usefulness of novel denoising process for depicting mCNV. This study included 20 eyes of 20 patients with mCNV. Ten en face images taken in a 3 × 3 mm macular cube were obtained from outer-retina-to-choriocapillaris layer. Three image types were prepared for analysis; single images before and after the denoising process accomplished deep learning (single and denoising groups, respectively) and up to 10 images were averaged (averaging group). Pairwise comparisons showed vessel density, vessel length density, and fractal dimension (FD) were higher; whereas, vessel density index (VDI) was lower in single group than in denoising and averaging groups. Detectable CNV indices, contrast-to-nose ratio, and CNV diagnostic scores were higher in denoising and averaging groups than in single group. No significant differences were detected in VDI, FD, or CNV diagnostic scores between denoising and averaging groups. The denoising process can utilise single OCTA images to provide results comparable to averaged OCTA images, which is clinically useful for shortening examination times with quality similar to averaging.

## Introduction

Myopic choroidal neovascularisation (mCNV) is one of the commonest vision-threatening complications in highly myopic eyes^1^. Early mCNV treatment with an intravitreal anti-vascular endothelial growth factor (anti-VEGF) agent is safe and effective^2–4^. Anti-VEGF therapy has become the standard-of-care and recommended first-line treatment option for mCNV^5^. Therefore, early detection of mCNV has become essential to prevent mCNV complications.

Optical coherence tomography angiography (OCTA) is a novel, noninvasive modality that facilitates depth-resolved visualisation of retinal and choroidal blood flow without a dye injection, which may induce general complications^6^. A previous study reported the usefulness of OCTA in detecting mCNV^7^. Furthermore, OCTA-derived parameters of vessel length density (VLD) and fractal dimension (FD) can be predictors of poor visual outcomes after anti-VEGF therapy in mCNV^8^. Recently, some reports showed that averaging multiple en face OCTA images improves image quality^9–12^. However, it takes longer to obtain many OCTA images for averaging than to take a single OCTA image. A novel OCTA device with denoising process function accomplished by deep learning that has been used for clinical practice^13^ can clearly visualise OCTA images. However, the reliability of denoised OCTA images has not been shown in mCNV.

This study compares single OCTA mCNV images from before and after the denoising process and results of averaging multiple images to assess whether the denoising process was clinically useful for depicting mCNV.

## Methods

This prospective, observational, case-series study was approved by the ethics committee of the Kyoto University Graduate School of Medicine (Kyoto, Japan). All study protocols adhered to the tenets of the Declaration of Helsinki and its later amendments. The nature of the study and possible risks and benefits of participation were explained to all study candidates. All individuals who agreed to participate provided written informed consent.

### Participants

This study included consecutive patients with highly myopic eyes (defined by a spherical equivalent ≤ −6.00 dioptres and/or an axial length (AL) ≥ 26 mm) with mCNV, who visited the Department of Ophthalmology and Visual Sciences at Kyoto University Graduate School of Medicine (Kyoto, Japan) between November 2017 and September 2019. All patients underwent a comprehensive ophthalmologic examination including autorefractometry, measurement of best-corrected visual acuity with a decimal visual acuity chart (Landolt chart), AL measurement by partial coherence interferometry (IOLMaster, Carl Zeiss Meditec, Inc, Dublin, CA), indirect ophthalmoscopy, slit-lamp biomicroscopy, colour fundus photography (TRC-NW8F; Topcon Corp, Tokyo, Japan), spectral domain optical coherence tomography (SD-OCT; Spectralis HRA + OCT; Heidelberg Engineering, Heidelberg, Germany), fundus fluorescein angiography (FA), fundus indocyanine green angiography (ICGA; HRA II; Heidelberg Engineering), and OCTA (OCT-HS100; Canon Lifecare Solutions, Inc, Tokyo, Japan) with a maximum 10 images being averaged; this image maximum is more than the images averaged (5 images) in a previous study^12^. Retinal specialists diagnosed mCNV using SD-OCT, FA, and ICGA data. The inclusion criteria were a clinical diagnosis of mCNV and mCNV presence determined using averaged OCTA images by a retinal specialist (MM). The exclusion criteria were OCTA images of poor quality that hindered image analysis including significant segmentation errors and motion artefacts and the presence of other eye diseases including epiretinal membrane and vitreomacular traction syndrome. When both eyes of a patient were eligible, the right eye was selected for analysis.

### Quantitative assessment of OCTA images

**Figure 1:**
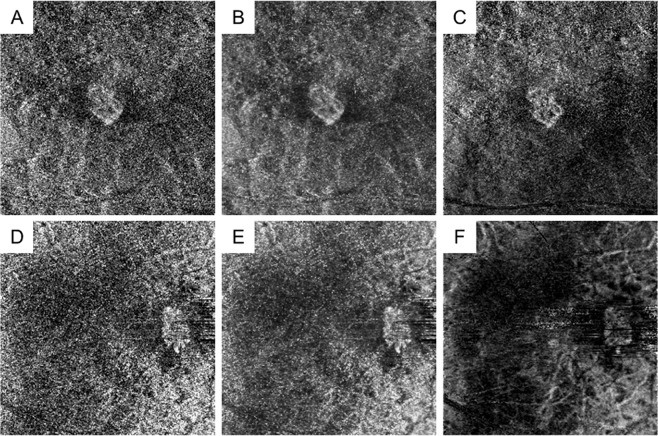
Representative optical coherence tomography angiography (OCTA) images of myopic choroidal neovascularisation (mCNV) after denoising and averaging processes. Images of a 76-year-old female patient (**A–C**) and a 60-year-old female patient (**D–F**) with mCNV in the right and left eyes, respectively. (**A,D**) Single OCTA images before the denoising process are shown. (**B,E**) Single OCTA images after the denoising process are shown. (**C,F**) OCTA images after the averaging process with a maximum of 10 single images are shown. The denoising process allowed for mCNV visualisation with a high contrast.

All OCTA images were acquired using the macular cube (3 × 3 mm) protocol of the OCT-HS 100 scanner containing 232 × 232 A-scans. The built-in software automatically performed layer segmentation; the en face image of outer-retina-to-choriocapillaris (ORCC) layer was obtained with the inner and outer boundaries at the outer border of the outer plexiform layer and 8 μm beneath the Bruch’s membrane, respectively, as previously reported^14^. Three types of OCTA images were prepared for analysis in each participant (Fig. 1): (1) the first single image before the built-in denoising process was accomplished by deep learning with image augmentation (detailed information is undisclosed) undertaken with the built-in software of OCT-HS100 (single group), (2) the first single image after the denoising process (denoising group), and (3) averaged images from a maximum of 10 images (averaging group). When the first single image quality was poor, the second or third single image was selected.

**Figure 2:**
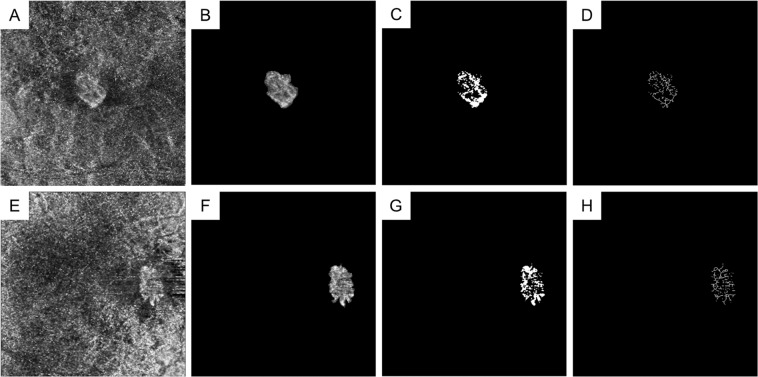
Representative optical coherence tomography angiography (OCTA) images of myopic choroidal neovascularisation (mCNV) before and after cropping, binarisation, and skeletonisation. Images of a 76-year-old female patient (**A–D**) and a 60-year-old female patient (**E–H**) with mCNV in the right and left eyes, respectively. (**A,E**) Original single OCTA images before the denoising process are shown. (**B,F**) Cropped OCTA images demonstrating the neovascular lesion are shown. (**C,G**) Binarised images, used for calculating the vessel density (VD), are depicted that show the entire neovascular lesion. (**D,H**) Skeletonised images were used to calculate the vessel length density (VLD) and fractal dimension (FD). Vessel diameter index (VDI) values were calculated using VD and VLD. (**A–D**) The values of VD, VLD, VDI, and FD were 34.1%, 11.5%, 31.1, and 1.5, respectively. (**E–H**) The VD, VLD, VDI, and FD values were 37.3%, 13.9%, 28.3, and 1.6, respectively.

In OCTA en face images of the ORCC layer, the parameters of vessel density (VD), VLD, vessel diameter index (VDI), and FD that represent the CNV status were measured for images from individuals in the 3 groups using open-source software (ImageJ, National Institutes of Health, Bethesda, MD) in accordance with a previous report^8^. Briefly, once the CNV lesion was manually cropped, the VD was defined as the ratio (%) of the area occupied by vessels after binarisation by local thresholding with the Otsu method. The VLD value was defined as the ratio (%) of the area occupied by skeletonised vessels after skeletonisation (Fig. 2)^9,15,16^. The VDI value was calculated using the following formula:

$${\rm{VDI}}=({\rm{VD}}/{\rm{VLD}})\times {\rm{Littmann}}\mbox{'}{\rm{s}}\,{\rm{coefficient}},$$

where VDI represents the average vessel calibre. FD was calculated by measuring the degree of pattern complexity by the box-counting method that determines vessel complexity, as described previously^15,17^. The absolute value of VDI required Littmann’ correction^18^. These OCTA-derived parameters would demonstrate the visible vascularity of CNV.

### Detectability indices of choroidal neovascularisation

**Figure 3:**
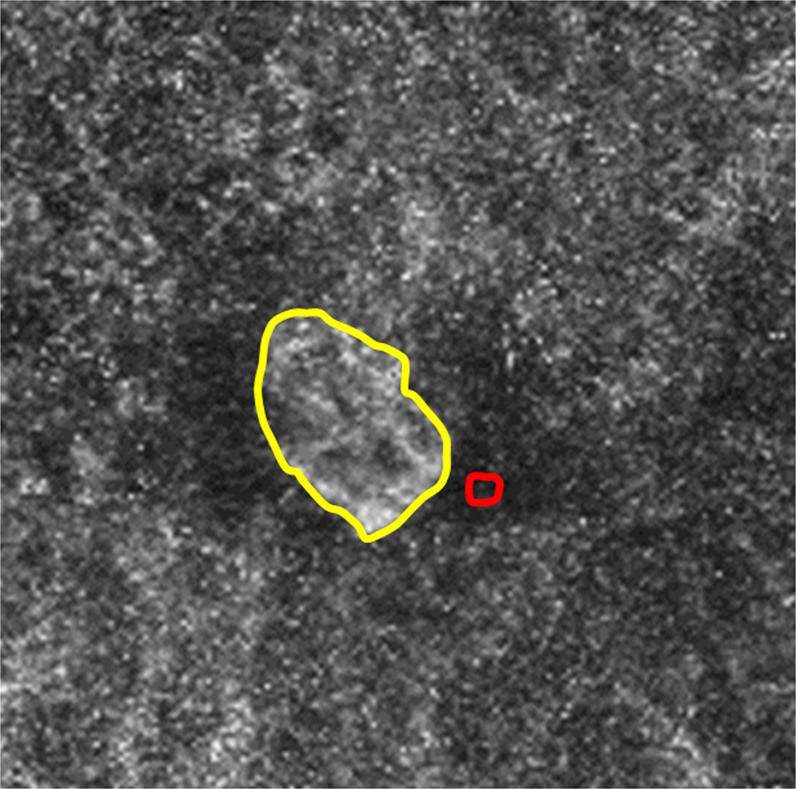
A representative image for selecting the foreground and background areas. Image of a 76-year-old female patient with myopic choroidal neovascularisation (mCNV) in the right eye. Foreground (within the yellow line) and background (within the red line) areas were selected to calculate the contrast-to-noise ratio (CNR). The darkest area around the CNV was set as the background and the whole CNV area was set as the foreground (CNR 1.6).

To evaluate CNV detectability indexes, we assessed the contrast-to-noise ratio (CNR) and CNV diagnostic scores for images of three groups. Although the foveal avascular zone is generally used as the background in CNR analyses for vascular flow in the superficial and deep capillary plexus layers^11^, avascular sites such as the foveal avascular zone are absent in the ORCC layer. Therefore, the darkest area around the CNV area was designated as the background and the whole CNV area was defined as the foreground (Fig. 3). The same background and foreground sites were selected in 3 individual images. Mean grey values and their standard deviations were analysed using ImageJ and the CNR was calculated as follows:

$${\rm{CNR}}=(f-b)/\sqrt{{{\delta }_{f}}^{2}+{{\delta }_{b}}^{2}},$$

where f and b are the mean grey values of the foreground and background, respectively, and δ_f_ and δ_b_ are their standard deviations, respectively. When the CNR is high, the CNV area has a higher contrast than that of the area around it; thus, a CNV area with a high CNR is thought to be more detectable than a CNV area with a low CNR.

Two blinded retinal specialists (YK and SK) individually and subjectively randomly assessed CNV presence without information whether mCNV was present. The subjective assessments consisted of CNV diagnostic scores that were categorised as follows: 0 = no CNV, 1 = CNV suspicion, and 2 = CNV presence. When the investigator (MM) found that each score differed considerably between the 2 investigators, a reassessment was conducted until the difference of scores fell within 1. Thus, the mean scores from 2 investigators were used for analysis. Taken together, the CNV diagnostic scores imply total subjective judgement of CNV presence from factors including visible vascularity of CNV (OCTA-derived parameters) and differences between CNV and its surroundings (CNR).

### Sub-analysis of measurement time

The time that a healthy person fixes his/her face on the OCTA device during a single scan and 10 scans for 1 eye as well as the time required for the denoising process and averaging after scanning were measured.

### Statistical analysis

Data are presented as the mean ± the standard deviation where applicable. All statistical analyses were undertaken in using SPSS (version 21; IBM, Armonk, NY). Statistical comparisons among the 3 groups were conducted using an analysis of variance with repeated measures; subsequent pairwise comparisons were undertaken with a Bonferroni test. *P*-values < 0.05 were considered statistically significant.

## Results

Twenty eyes of 20 patients met the inclusion criteria (15 women; 66.9 ± 12.4 years). The AL and refractive errors of all patients were 28.41 ± 1.27 mm and −6.66 ± 4.67 dioptres, respectively. Nine patients (45%) had undergone intraocular lens implantation after cataract surgery, although the remainder of patients had not. There were significant differences in VD, VLD, VDI, FD, CNR, and CNV diagnostic scores among the 3 groups (Table 1). Pairwise comparisons in OCTA-derived parameters show that VD, VLD, and FD were higher in the single group than in the denoising and averaging groups. The VDI was lower in the single group than in the denoising and averaging groups. With regard to CNV detectability indices, CNR and CNV diagnostic scores were higher in the denoising and averaging groups than in the single group. There were no significant differences in VDI, FD, or CNV diagnostic scores between the denoising and averaging groups. Data for these comparisons are described in Table 1.

**Table 1:** Comparison of parameters analysed from optical coherence tomography angiography images.

	Single group (G1)	Denoising group (G2)	Averaging group (G3)	*P*-values among the 3 groups	*P*-value of pairwise comparisons		
					G1 vs G2	G1 vs G3	G2 vs G3
VD, %	33.4 ± 8.3	29.7 ± 9.3	26.0 ± 12.1	<0.001*	<0.001*	<0.001*	0.004*
VLD, %	11.9 ± 2.9	9.0 ± 3.0	7.7 ± 3.8	<0.001*	<0.001*	<0.001*	0.03*
VDI	31.7 ± 5.1	37.6 ± 6.7	40.2 ± 9.9	0.001*	<0.001*	0.002*	0.66
FD	1.5 ± 0.1	1.4 ± 0.1	1.3 ± 0.3	<0.001*	<0.001*	0.004*	0.61
CNR	1.2 ± 0.3	1.7 ± 0.4	1.5 ± 0.3	<0.001*	<0.001*	<0.001*	0.03*
CNV diagnostic scores	0.8 ± 0.6	1.3 ± 0.6	1.6 ± 0.4	<0.001*	0.008*	<0.001*	0.12

Data are presented as the mean ± the standard deviation.

VD = vessel density; VLD = vessel length density; VDI = vessel diameter index; FD = fractal dimension; CNR = contrast-to-noise ratio; CNV = choroidal neovascularization.

Statistical comparisons were conducted among the three groups using an analysis of variance with repeated measures; subsequent pairwise comparisons were conducted with a Bonferroni test.

*Statistically significant *P* < 0.05.

The time that a 28-year-old healthy female individual fixed her face on the OCTA device at a single scan for 1 eye was approximately 5 seconds and the time for 10 scans was approximately 248 seconds, although the time would vary in accordance with individual patient status. The actual time of the denoising and averaging processes after scanning was 5 and 24 seconds, respectively.

## Discussion

The present findings demonstrate that the built-in denoising process accomplished by deep learning improved the quality of single OCTA images of mCNV in all OCTA-derived parameters and CNV detectability indices in this study; furthermore, single denoised OCTA images could be used in lieu of averaged images. As it takes longer to obtain OCTA images via the averaging process than to obtain single images, the denoising process can decrease the burden on patients and examiners as it only requires 1 image.

In the present study, denoising accomplished by deep learning lowered VD, VDI, and FD, but increased VLD in mCNV; these trends are similar with the averaging results. Moreover, on OCTA images of mCNV, CNV vessels became sharp and had low pattern complexity after the denoising and averaging processes. As a result, the denoising and averaging processes presented CNV vessels as distinct from normal vessels. Thus, the denoising process is useful for detecting mCNV using a single OCTA image.

Biomicroscopic features of mCNV include a light-coloured lesion with a dark and hyperpigmented rim^19^. Therefore, the CNV detectability index included CNR in the present study with the CNV area as the foreground and the darkest sites around the CNV area as the background. The CNR values were higher in the denoising group than in the single and averaging groups. The OCTA images of mCNV after the denoising process had a high contrast between the CNV area and the dark rim around the CNV area. Furthermore, CNV diagnostic scores were higher in the denoising group than in the single group; there were no significant differences between the denoising and averaging groups. Thus, in clinical practice for mCNV detection, a denoising process is useful in a manner similar to that of averaging.

Artificial intelligence with deep learning technology has recently played an important role in diagnosis and treatment, health management, drug research and development, and precision medicine^13^. In ophthalmology, some researchers report that deep learning can support an automatic diagnosis of glaucomatous optic neuropathy, diabetic retinopathy, and central serous chorioretinopathy^20–22^. Furthermore, nonperfused areas could be automatically detected using deep learning^23^. In the present study, mCNV on single OCTA images could be depicted more clearly using an automatic denoising process that was accomplished by deep learning than by single OCTA images before the denoising process. In the future, deep learning technology will likely spread to various operations in ophthalmology.

The present study has some limitations. First, the sample size was small. Eleven eyes of 11 patients with averaged OCTA images of poor quality could not be analysed and were excluded. Improvements to OCTA devices are necessary in parallel with improvements in deep learning technology. Second, the cropping procedure of the CNV area using ImageJ was manually executed. However, because the CNV border is often clear in mCNV, a previous report also used a manual cropping procedure^8^. Third, the background for the CNR assessment was manually selected; we could not automatically select the whole area while excluding the CNV area. However, due to the mean grey value of the background included in normal vessels, the value was inadequate to distinguish the dark rim around the mCNV region^19^. To avoid bias, the same sites were selected in all 3 groups in each participant. Fourth, since denoised mCNV OCTA images are altered (not original), the possibility that the images might not appear to be true CNV images was a concern. However, the denoising process did not produce any unexpected images. Fifth, 2 investigators did not assess CNV diagnostic scores of OCTA images without mCNV. Thus, there may have been a bias in the CNV diagnostic scores.

In conclusion, a novel built-in denoising process accomplished by deep learning can create single OCTA images similar to that of averaged OCTA images. This process is clinically useful to shorten examination times whereas providing results of similar quality as obtained with averaging.

## Data Availability

All data generated or analysed during this study are included in this published article.

## References

[1] Hampton GR, Kohen D, Bird AC (1983). Visual prognosis of disciform degeneration in myopia. Ophthalmology.

[2] Lai TY, Luk FO, Lee GK, Lam DS (2012). Long-term outcome of intravitreal anti-vascular endothelial growth factor therapy with bevacizumab or ranibizumab as primary treatment for subfoveal myopic choroidal neovascularization. Eye (Lond)..

[3] Sarao V, Veritti D, Macor S, Lanzetta P (2016). Intravitreal bevacizumab for choroidal neovascularization due to pathologic myopia: long-term outcomes. Graefes Arch. Clin. Exp. Ophthalmol..

[4] Chhablani J (2018). Intravitreal bevacizumab monotherapy in myopic choroidal neovascularisation: 5-year outcomes for the PAN-American Collaborative Retina Study Group. Br. J. Ophthalmol..

[5] Ohno-Matsui K, Ikuno Y, Lai TYY, Gemmy Cheung CM (2018). Diagnosis and treatment guideline for myopic choroidal neovascularization due to pathologic myopia. Prog. Retin. Eye Res..

[6] Yannuzzi LA (1986). Fluorescein angiography complication survey. Ophthalmology.

[7] Miyata M (2016). Detection of myopic choroidal neovascularization using optical coherence tomography angiography. Am. J. Ophthalmol..

[8] Hosoda Y (2018). Novel predictors of visual outcome in anti-VEGF therapy for myopic choroidal neovascularization derived using OCT angiography. Ophthalmol. Retina.

[9] Uji A (2017). Impact of multiple en face image averaging on quantitative assessment from optical coherence tomography angiography images. Ophthalmology.

[10] Uji A (2017). Choriocapillaris imaging using multiple en face optical coherence tomography angiography image averaging. JAMA Ophthalmol..

[11] Uji A (2018). Multiple enface image averaging for enhanced optical coherence tomography angiography imaging. Acta Ophthalmol..

[12] Murakawa S, Maruko I, Kawano T, Hasegawa T, Iida T (2019). Choroidal neovascularization imaging using multiple en face optical coherence tomography angiography image averaging. Graefes Arch. Clin. Exp. Ophthalmol..

[13] Xu J, Xue K, Zhang K (2019). Current status and future trends of clinical diagnoses via image-based deep learning. Theranostics..

[14] Roisman L (2016). Optical coherence tomography angiography of asymptomatic neovascularization in intermediate age-related macular degeneration. Ophthalmology.

[15] Reif R (2012). Quantifying optical microangiography images obtained from a spectral domain optical coherence tomography system. Int. J. Biomed. Imaging..

[16] Kim AY (2016). Quantifying microvascular density and morphology in diabetic retinopathy using spectral-domain optical coherence tomography angiography. Invest. Ophthalmol. Vis. Sci..

[17] Landini G, Murray PI, Misson GP (1995). Local connected fractal dimensions and lacunarity analyses of 60 degrees fluorescein angiograms. Invest. Ophthalmol. Vis. Sci..

[18] Littmann H (1992). Determination of the true size of an object on the fundus of the living eye. By H. Littmann from the original article, “Zur Bestimmung der wahren Grosse eines Objektes auf dem Hintergrund des lebenden Auges”, which originally appeared in Klinisches Monatsblätter für Augenheilkunde 1982; 180:286–9. Translated by TD Williams. Optom. Vis. Sci..

[19] Cheung CMG (2017). Myopic choroidal neovascularization: review, guidance, and consensus statement on management. Ophthalmology.

[20] Liu, H. *et al*. Development and Validation of a Deep Learning System to Detect Glaucomatous Optic Neuropathy Using Fundus Photographs. *JAMA Ophthalmol*, 10.1001/jamaophthalmol.2019.3501. (2019). 10.1001/jamaophthalmol.2019.3501 PMC6743057 31513266

[21] Nielsen KB, Lautrup ML, Andersen JKH, Savarimuthu TR, Grauslund J (2019). Deep learning-based algorithms in screening of diabetic retinopathy: a systematic review of diagnostic performance. Ophthalmol. Retina.

[22] Zhen, Y. *et al*. Assessment of central serous chorioretinopathy depicted on color fundus photographs using deep learning *Retina*. 10.1097/IAE.0000000000002621. (2019). 10.1097/IAE.0000000000002621 31283737

[23] Nagasato D (2019). Automated detection of a nonperfusion area caused by retinal vein occlusion in optical coherence tomography angiography images using deep learning. PLoS ONE.

